# Rediscovering the ‘Meaning of Science’? Hans Morgenthau and the ethics debate in quantum IR

**DOI:** 10.1177/00471178241265635

**Published:** 2024-07-25

**Authors:** Michael PA Murphy

**Affiliations:** Queen’s University & Centre for International Governance Innovation

**Keywords:** epistemology, ethics, Hans Morgenthau, International Relations Theory, quantum IR, science

## Abstract

The concept of science has long played an important role in defining the field of international relations, both in its broader epistemological debates and in the formation of distinct research traditions. I argue that the emerging quantum approaches to international relations theory destabilize the conventional bifurcations of scientific and humanistic approaches to international relations, and that paying attention to this uncertainty can help build a broader understanding of not only quantum international relations but the field as a whole. Through a close reading of Hans Morgenthau’s commentaries on science in *Scientific Man Versus Power Politics* and *Science: Servant or Master*, this article argues that the ‘ethics objection’ that Morgenthau leveled against scientism is in fact overcome by quantum international relations.

## Introduction

‘*The traditional concepts of time, space, and the law of gravitation have succumbed to the theory of relativity; the quantum theory has transformed causation into statistical probability and replaced determinism by the principle of indeterminacy. What scientist philosophy and, under its influence, nineteenth-century political thought and the social sciences refer to as their object of emulation is a ghost from which life has long since departed*’.^1^

In *Scientific Man Versus Power Politics*, Hans Morgenthau’s rebuke of liberalism and scientism in international politics draws directly on the findings of quantum mechanics to critique the false certainty that scientism offered social scientists. Not anti-science but carefully anti-scientism, Morgenthau highlights that the claims to certainty that scientistic beliefs of social science permitted were clearly at odds with modern theories of natural science that were more at home in uncertain worlds. The rise of quantum theory and relativity – among other complex models – replaced certainty with probability. And yet, as Morgenthau noted, scientistic claims of certainty abounded in social sciences. This false certainty of social science led to a whole panoply of ethical and political failings to which Morgenthau would spend his career responding. This article picks up Morgenthau’s connection to quantum theory,^2^ not to present Morgenthau as a justification for the superseding of Newtonian IR by quantum IR, but to question if contemporary quantum approaches to International Relations run afoul of Morgenthau’s objection to scientistic thinking.

The emerging research community of quantum international relations is a pluralistic group that includes a variety of methods for linking international relations with conceptual tools emerging from quantum theory. In the context of their introductory essay, James Der Derian and Alexander Wendt frame a recent special issue of *Security Dialogue* in recognition of precisely this multiplicity by saying that ‘we offer quantum approaches (in the plural) rather than propose a quantum theory (in the singular) of international relations’.^3^ Indeed, any review of the literature in quantum international relations includes a wide range of approaches to transcending disciplinary divides, from consciousness to war and diplomacy,^4^ social theory to social change.^5^ The subjects range from metatheoretical concerns of causality^6^ to empirical considerations of surveillance and language.^7^ This intellectual diversity has permitted a wide range of theoretical engagements within a relatively short period of active scholarly publication.

However, the position of quantum international relations within broader theoretical debates in international relations is not immediately clear. Thoughtful critics of quantum approaches have asked if the cost of integrating complex and potentially alienating vocabulary into international relations produce sufficient benefit, if similar theoretical conclusions can be reached through the combination of existing concepts.^8^ To reach thoughtful but skeptical readers, it is imperative that quantum international relations reflect seriously on the question of why the context and contribution are not more readily apparent. I argue that the specific engagement with science that exists in quantum international relations disrupts conventional assumptions about a science/ethics bifurcation and aligns with Morgenthau’s notion of the ethics/epistemology relationship that he found sorely lacking in scientistic paradigms.

New quantitative methodologies, systemic and process-based explanations, and behavioral models swept into a field of international relations previously dominated by lawyers, historians, theorists, and practitioners. While shorthand labels of ‘behavioral versus traditional’ or ‘qualitative versus quantitative’ are sometimes applied to the belligerents, there is so much variation within each party that the accuracy of any generalization is limited.^9^ Nevertheless, a pivotal moment in the conventional narrative of international relations is understood to be the debate between humanistic approaches and social scientists. While in (American) political science, the victory of the behavioralists was declared openly by Robert Dahl,^10^ commentators on international relations were hesitant to overstate the success of the movement.^11^ Proponents of behavioralism were not shy in staking out the benefits of borrowing quantitative approaches from economics and beyond. J. David Singer, for instance, argued that ‘a behavioral science approach might lead to fuller knowledge and more powerful theory’ before extending to say that ‘the payoffs need not end with the intellectual and the scholarly’, noting the utility of predictive models for policy questions.^12^ Traditionalists disagreed, perhaps none more strongly than Hedley Bull, who argued that the scientific approaches were weakened by their ignorance of classical insights and ‘fetish for measurement’^13^; ultimately, Bull argued that the insights produced by scientific international relations could readily be readily incorporated within a classical approach that did not eschew the insights of history, philosophy, ethics, law, and other fields.^14^ Hans Morgenthau similarly argued against the limitations that the so-called scientific approaches placed upon the depth of analysis: ‘in such a theoretical scheme, nations confront each other not as living historic entities with all their complexities but as rational abstractions, after the model of “economic man,” playing games of military and diplomatic chess according to a rational calculus that exists nowhere but in the theoretician’s mind’.^15^ The behavioralists would respond to these criticisms of the limitation of scientific thinking on multiple fronts. Morton Kaplan, for example, argues both that traditionalists misrepresent the logic of scientific analysis in their critique, and furthermore that they inflate the philosophical rigor of their own approach.^16^ The behavioral revolution left an indelible mark on the field, with Stanley Hoffmann calling IR an ‘American Social Science’^17^ and Kal Holsti arguing that the introduction of these self-styled scientific approaches ‘*has* fundamentally rearranged the methods used to obtain knowledge’.^18^ The imprint continues to structure the field in many ways, with bifurcations into explaining and understanding, rationalism and reflectivism, and positivism and post-positivism^19^ all bearing an at least familial resemblance to the second great debate.

Morgenthau’s epistemological critique of scientism developed through a pair of thought-provoking texts separated by decades of the discipline’s development. Although his earlier *Scientific Man and Power Politics*^20^ is better-known, I argue that Morgenthau’s *Science: Servant or Master?* is notable because of his consideration of both technical aspects of scientific developments as well as a rigorous philosophical essay reflecting on the meaning of science.^21^ A concern with the influence of science and scientism on the practice and theory of international relations spanned Morgenthau’s writings from first to last,^22^ and it is this extended meditation on science as an idea that provides Morgenthau’s final word on the matter. Morgenthau castigates the scientistic attitude that separates philosophical thinking about morality and humanity from the rationalistic positivism that – guided by an ideology of scientific progress – eschews the work of theoretical thinking that is necessary to inform political life. Not only is the work notable for the quality of its reflections but as an artefact that continues the work of *Scientific Man Versus Power Politics*, this collection connects the consideration of science and ethics to the heart of the conventional history of international relations.

Morgenthau’s opening essay in that volume is significant for quantum International Relations not merely because of his place in the canon of the field or his enduring concern with science in his corpus. Rather, it is because the emergent ‘ethics debate’ in quantum international relations overcomes his specific metatheoretical critique of scientistic thinking developed through *Science: Servant or Master*. If quantum international relations is able to overcome Morgenthau’s strong critique of scientism, then it stands to reason that well-worn worries and scientific skepticisms can be placed aside in favor of a more open reading of quantum international relations scholarship. The remainder of this article proceeds in four sections. The first contextualizes *Science: Servant or Master* in Morgenthau’s work before undertaking a close reading of the opening essay of that volume. The second section introduces the emergent ethics debate in quantum international relations, including the entangled ontology of Karen Barad. The third section brings quantum IR into conversation with Morgenthau’s ‘ethics objection’ to demonstrate how this emergent ethics debate passes the test that Newtonian social science may have failed. The conclusion explores the implications of quantum international relations overcoming Morgenthau’s ethics objection and considers the implications for further research within and beyond quantum IR. Given the article’s primarily metatheoretical focus, I have broken with the standard practice of defining all quantum terms in detail; instead, this article uses the minimal necessary jargon with explanatory footnotes where technical details are materially significant to the content of the claim.

## Morgenthau, scientism, and the ‘ethics objection’

An émigré from Nazi Germany to the United States, Hans Morgenthau was trained first as a lawyer, but drew on a variety of disciplines for inspiration – history, philosophy, sociology, and elsewhere – in his decades as a leading commentator on foreign policy and international relations theory. In the conventional narratives of international relations, Morgenthau sits in a central position on the Realist Mount Rushmore. Yet, this risks reductionism; although popularly reduced to a stand-in for classical realism, commentary since 2000 has helped to recognize Morgenthau as a complex thinker and an ‘uneasy realist’.^23^ Morgenthau’s writings are also infused with a traditionalist epistemology that accords high value to the role of ethics in knowledge production. Of particular interest for the purposes of this article are careful distinctions that he makes between the follies of scientism and rationalism, on the one hand, and the utility of science and rationality, on the other.^24^ As Felix Rösch has argued previously, Morgenthau’s epistemology sees the normative and ethical aspects of human interaction as essential to a fulsome understanding of political life.^25^ While certainly recognized for his contributions to international relations theory *writ large*, the depth of his engagement on the question of science has not been fully appreciated. To present what I call Morgenthau’s ‘ethics objection’ to scientism, this section begins with a discussion of *Scientific Man Versus Power Politics* before turning in detail to ‘The Meaning of Science’ essay from *Science: Servant or Master*.^26^

Like E.H. Carr’s *Twenty Year’s Crisis* a decade earlier, Morgenthau’s primary object of critique in *Scientific Man Versus Power Politics* is the failure of liberal approaches to international relations.^27^ Both catalog the failures of the theory of the harmony of interests, although Carr’s rebuke of utopianism is replaced by Morgenthau with a specific critique of the way that scientific rationalism misjudged the humanity of international relations.^28^ In contrast to Morgenthau’s tripartite model of humanity as biological impulses and spiritual aspirations in addition to rational processes, scientism ‘misconstrues the function reason fulfils’ by equating with it the entirety of human thought.^29^ This assumption of a ‘Scientific Man’ reframes the purpose of political considerations. No longer is politics an art to be considered by a statesman, but instead a rational calculus. It is this rationalist reductionism which facilitates the emergence of utopian thought:

Social problems, then, are very much like mathematical problems. They can all be solved and their solution is implicit in the very essence of reason from which it is to be evolved through a chain of logical deductions. No wonder, then, that this philosophy has an essentially optimistic outlook. Since man has the faculty of attaining perfection in reason, he has also the faculty of attaining perfection in goodness and success.^30^

In a world of scientism, ethical questions of human flourishing become functionally equivalent to technical questions of measurement and prediction: ‘the ethical perfection of utilitarian rationalism consists simply in acquiring the empirical knowledge of how certain effects are coordinated with certain actions, that is, what good, in utilitarian terms, to expect from certain actions’.^31^ This radically limits the scope of functions undertaken in the study of international politics; no longer are the base needs or lofty dreams considered, but only the rational calculations that can be understood through the philosopher’s stone of scientific inquiry.^32^ In place of a careful analysis of the ethical stakes of humanity in all their complexity, scientism examines only a limited explanation and effaces any detailed consideration of morality.^33^ We can see here already the important link between epistemology and ethics, as the narrowness of scientistic epistemology removes ethics from the scope of knowledge production. In addition to inscribing a moral failing, this epistemology also sidelines an important aspect of human life.

Of particular concern to Morgenthau is the erasure of any ability of a scientistic epistemology to explore ethics and power. These are very much connected considerations for Morgenthau, who sees ethics and power as essential elements of human being – rather than the accident of a particular scientific method.^34^ Other ethical codes may be discussed, but a utilitarian calculus of policy efficiency entails a radical reduction of all means to their measurable ends.^35^ This empties the ethical content of how political scientists and practitioners seek to understand the ethical dilemmas, moral quandaries, and rock-and-a-hard-place decisions that necessarily exist in the world. ‘There is no escape from the evil of power’, Morgenthau tells us,^36^ and scientistic views of the world as rational mechanisms for technical manipulation only redoubles in its inability (through the erasure of meaningful ethics) to understand power. If, as described above, rationalism reduces ethics to a question of technical optimization of causes and effects, there is no room to question ethical stakes as such. Existence is proof of justification within the scientistic paradigm, not because of any moral reasoning but because any other form of reasoning has been removed, and ‘the quest for and the defense of power then become aberrations from the scientific attitude, which looks for causes and remedies’.^37^ The lesson of the failure of the harmony of interests is that the choice to ignore power politics does not mean that power politics ignores those societies pursuing technical questions. By ignoring ethics, scientism loses the ability to understand and guide actions in the realm of power politics. Thinkers promoting a liberal-rationalist utopia not only fail in terms of political practice – as the League of Nations demonstrated – but also in theory through their inability to meaningfully assess ethical matters or weigh ethical considerations. As Molloy has argued, Morgenthau does not believe that political truth can be reached by subsuming concerns of power and attention to ethics under scientific methods.^38^ This provides a *prima facie* form of the ethics objection, a ground-level analysis that if science and ethics are separated, then the scientific analysis is incomplete. While this will return below as our zero-level interaction between the quantum ethics debate and Morgenthau’s ethics objection, we find further details about the specific content of the ethics objection by turning our attention to another work. While this close reading of *Science: Servant or Master* takes a great deal of our time, care in fully describing the ethics objection at present will allow for a clearer measurement of the quantum ethics debate later on.

In contrast to *Scientific Man Versus Power Politics*, the hierarchy of liberalism and scientism as objects of critique are inverted in *Science: Servant or Master*. The first sets its sights on the political project of liberalism informed by the ‘Scientific Man’ worldview, whereas the latter takes aim at scientism directly for its ethical and epistemological failure. *Science: Servant or Master* approaches the relationship between science and international relations in two different ways. First, in the opening essay ‘The Meaning of Science’, Morgenthau employs a philosophical approach, exploring the relationship between the idea of science and the study of politics. Science here is defined broadly, as ‘the attempt to make experience conscious in reason in a theoretically valid, systematic way’.^39^ In the latter two chapters, Morgenthau instead discusses the technical and empirical impact of developments in the natural sciences – first in the empowerment of bureaucracy versus popular government and then in the shifting conditions produced by the atomic age. These definitions are narrower in conceptual scope, and lead to a different tone than the exploratory first essay; however, they establish that science serves as Morgenthau’s primary concern in the work as a whole.

A reader of ‘The Meaning of Science’ familiar with Morgenthau’s earlier work may first be struck with the coherence of this essay with the discussion in *Scientific Man Versus Power Politics*. In large part, Morgenthau’s critique of science as it interacts with international relations revives the argument that the scientistic epistemology must be found lacking as a guide for politics. He laments that ‘what these social sciences aim at is not so much to inform the will of the statesman as to replace it’ by their own calculations, justified by the overarching assumption that ‘each social problem is supposed to be soluble by the one rational solution, scientifically determined’.^40^ The adoption of a hyperspecialized vocabulary and set of analytical techniques by scientistic approaches to inquiry seeks to reinforce this domination of politics by social sciences. Morgenthau argues that ‘much of the social sciences ends up in a kind of secularized Talmudism, an afunctional social game with methodology and terminology, accessible only to the initiated’ to the extent that methodology dictates what kinds of puzzles take priority, producing aesthetically appealing but ultimately random and unsystematic knowledge.^41^ This epistemological reductionism of the world to the problem set disserves humanity, as ‘politics, formerly a struggle of interests defined in terms of power, is reduced to a demonstration of the truths that the social sciences have to offer for the solution of political problems’.^42^ The surface-level failure is the detachment and elitism of a social science detached from society, but the specter of power politics lingers. Indeed, part of the danger for a state falling for the ideology of scientism is that the simplification of rationalism ‘appears as the very cause of the dangers it pretends to be able to prevent’.^43^ Scientistic reductionism is vulnerable to the crushing force of power politics, despite – and perhaps even *especially because of* – the rhetorical strength of its rationalistic narrative.

Yet, this practical critique that scientism provides incomplete and inadequate advice is not all that is familiar. Indeed, Morgenthau again rebukes the reductionism of the scientistic approach for emptying its considerations of moral content, operating at two levels: ‘Science’, he tells us, ‘has not only lost its relation to a transcendent value from which it could receive its meaning but, more importantly, it has also lost the awareness of the need for such a transcendent orientation’.^44^ In other words, Morgenthau rebukes the practice of science for the sake of science for losing sight of the higher purpose of the moral and material betterment of mankind.^45^ While the first part of this problem follows logically enough from the rewriting of social inquiry from the perspective of scientific dogma rather than power politics, the divorce of social knowledge from questions of ethics bankrupts the enterprise, insofar as science ‘is no longer aware of the need for moral distinctions to be made within the sphere accessible to human knowledge’.^46^ We should be careful here to point out that Morgenthau does not state that scientific modes of knowledge production communicate no values; despite what proponents may argue, defining meaning from a scientific perspective implies a judgment of what is meaningful.^47^ However, there is a willful ignorance of the ethical prescriptions that flow forth from scientistic work. ‘When science was freed from the subjection to a heteronomous order of moral values’, Morgenthau tells us, ‘it established its freedom from any moral limits whatsoever’.^48^ The ignorance of ethical stakes is problematic in its own right, yet the mistake is only compounded because ignorance does not offer freedom from questions and implications of morality.^49^

But there is a third element to this ‘ethics objection’ that Morgenthau develops *contra* scientism; not only does he allege that scientistic approaches are empirically inadequate and morally inferior, but they are also *not truly scientific!* Morgenthau understands science to be a pursuit above the merely technical solving of problems for humanity’s ends, instead positioning it as a systematic and theoretical response to the ‘shock of wonderment’ that humanity experiences when confronted with the vastness and unknowability of the universe: ‘science is knowledge for man’s [sic] sake, for the sake of the shock of wonderment through which man encounters the world’.^50^ There is a necessary connection to be recognized here, as ‘science is the systematic, theoretical attempt to understand that which is empirically unknown yet knowable’, yet as scientific thinking pursues the unknown, science entails ‘the progressive loss of its empirical objectivity and . . . its transformation into philosophy’.^51^ Science that is curtailed within the domain of the empirically knowable is therefore incomplete and not *genuine science* for Morgenthau. This sentiment recalls the distinction that Morgenthau makes between quantum and relativity – which are open to uncertainty – and the abandoned scientific models of the 18th century that social scientists fetishize and emulate.^52^ Instead of the height of science representing intellectual problems to be solved through technical means, ‘the ultimate decisions that confront the scientific mind are, then, not intellectual but moral in nature’ and the core failing within the contemporary scientific paradigm is found in this disconnect between the philosophical connection of genuine science and the technical circumscription of contemporary science: ‘the refusal to make morally relevant use of that intellectual ability is the real deficiency of scientific man’.^53^ This careful argument is perhaps the most illuminating diagnosis of Morgenthau’s ethics objection – because science has lost its connection to philosophy and ethics, it is no longer genuine science.

Instead of genuine science, the name that Morgenthau gives to the truncated intellectual pursuit of the scientistic age is ‘rationalistic positivism’. We see in his description of this phenomenon that Morgenthau’s critique of scientific thought is not grounded in an opposition to science, but the technical truncation of scientism:

Rationalistic positivism has been unable to understand this subtle connection between science and philosophy. Fascinated by the success of the exact sciences in the empirical world, it has lost sight of the very instance of the unknowable. It conceived of science as systematic, theoretical knowledge of what was empirically knowable and of the universe as composed of objects, some already known, others not yet known, but as a matter of principle knowable and sooner or later sure to be known, dependent upon the progress of science.^54^

Morgenthau’s objection to scientistic epistemology tells us a great deal not only about the reverence that Morgenthau held for science as a field of inquiry, but also about how the failure of contemporary scientific practice – especially the scientific approaches to international relations – to live up to the vaulted ideals of science. *Science: Servant or Master* is careful to avoid the ‘two extremes of a quasi-religious faith in science and a quasi-religious disgust with science’ and instead probes the meaning of science in its broadest sense, questioning ‘what the true place of science is in the human scheme’.^55^ What I have here termed the ‘ethics objection’ to scientific thought in international relations is really a critique of the incomplete rational-positivist ideology that presented itself as science.

This review of Morgenthau’s two ‘science books’, with a particular focus on the opening essay of *Science: Servant or Master*, provides a rigorous theoretical foundation for understanding what the ethics debate can tell us about quantum international relations. Indeed, the objection that Morgenthau develops with regards to earlier scientific paradigms in international theory is largely – and despite the different foci between the two books – ethical in its impulse. While *Scientific Man Versus Power Politics* highlights the failure of scientific thinking in its utopian-liberal moment, it is the reductionism of scientism that leads to the fall. Similarly, when *Science: Servant or Master* calls attention to the incompleteness, vulnerability, and ignorance of scientistic approaches, all three elements rely on a meta-critique of the absence of ethical considerations. These works are situated within the context of different Great Debates, and take up arms against different competitors; however, the ‘ethics objection’ to scientistic perspectives remains constant.

## The ethics debate in quantum IR

The camp of quantum IR may share an intuition that quantum ideas are relevant for theoretical debates in International Relations, but the way that scholars engage with those ideas varies widely. Some ground their work in claims about quantum properties of physical reality while others draw weak associations of metaphors or analogies from quantum theory. Still other approaches borrow the mathematical formalism of quantum mechanics to redraw modeling or analysis of social phenomena, and others consider how Newtonian concepts embedded in the philosophical systems upon which IR theory draws might be replaced by alternative concepts from quantum mechanics. Participants in the ‘ethics debate’ in quantum IR have generally drawn from quantum social theory, often highlighting the work of Karen Barad, to suggest shifting from an ethics that tacitly assumes Newtonian assumptions about the world – for example, causal linearity, locality, ontological separability, lawlike regularity, epistemological objectivity – to a quantum set of assumptions – for example, relationality, uncertainty, paradox, entanglement, duality.This is a necessarily limited claim, as the greater utility of quantum concepts to explain some phenomena would imply that Newtonian ideas would be more apt in other contexts. The point is not that a quantum worldview magically offers omniscience, but that after a century of international relations abiding one set of limits, consideration of an alternative may provide valuable opportunities.

What is notable about the ‘ethics debate’ in quantum international relations in the context of Morgenthau’s rebuke of scientism as a reduction of humanity and ethics is just how central the roles of ethics, agency, and responsibility are in this philosophical reorientation. When boundaries are fuzzy and relationality is accorded ontological primacy, the ethics of entanglement supersede defenses based on unintended consequences or unforeseen events.^56^ The ethics debate in quantum international relations demonstrates the inextricability of ethical and political reflection.^57^ While the bulk of this section will focus on quantum international relations, I begin by offering a brief overview of Karen Barad’s approach to entangled ethics, which has been influential in the development of the ethics debate in quantum international relations. As we shall see, Barad’s contribution to the quantum ethics debate is significant to the comparison with Morgenthau because in their quantum theory, ethics are not a peripheral concern but fundamental to what it means to take on a quantum worldview.

The core of Barad’s philosophical project is a robust consideration of diffraction across multiple levels, to consider how experimental observations in quantum physics can ground an entirely new ethico-onto-epistemology. Significantly, this involves a move beyond ‘mere analogy’ to instead read social and political thought through quantum mechanics following a ‘diffractive methodology’.^58^ Barad is emphatic that *Meeting the Universe Halfway* is not an attempt to describe how some social phenomena are *like* quantum properties, but instead to reimagine the world based on the properties of diffraction. Instead of beginning with a concept of the object as a primary ontological unit, Barad argues that we must consider the *phenomenon* as a whole. While we may perceive bounded objects when we interact with them, the apparent boundaries of objects are created through out interactions.^59^ Their shift from a world of fixed objects to fuzzy phenomena suggests that relations are more ontologically significant than the relata, and any human activity – scholarly or otherwise – must be understood as action within a phenomenon through our relations.^60^ Barad therefore distinguishes ‘interaction’, a Newtonian concept where separate objects exert some kind of force on one another, from ‘intra-action’, a quantum concept where two related entities take on new properties through their continued relation within the system.

This radical priority of connectedness highlights the role of entanglement in Barad’s worldview. More than merely a term casually invoked to express connection, Barad is making an ontological claim that reality is composed of phenomena whose constituent relata come into being through their intra-action. Relata only emerge through their relations within a phenomenon (and cannot be understood beyond that relational context). Significantly, this connectedness is also a radically ethical proposition for Barad. As intra-actors within a phenomenon, Barad calls attention to ‘responsibility and accountability for the entanglements “we” help enact and what kinds of commitments “we” are willing to take on’.^61^ Coming to terms with our continued intra-action and relating within the phenomena we inhabit require us – in this worldview – to ‘meet the universe halfway’ by acknowledging a radical responsibility ‘to move towards what may come to be in ways that are accountable for our part in the world’s differential becoming’.^62^ The world as we experience and measure it in our personal and academic lives is one with which we are entangled and which we help to bring into being by our continued intra-action with our phenomenal surroundings.^63^

Barad’s work has inspired a variety of developments in international relations,^64^ and the ethics debate in quantum international relations places their approach to entanglement front and center. As noted in the introduction, the research community of quantum international relations includes a wide variety of strategies for engaging with quantum ideas. However, much of the work taking place around the question of quantum ethics follows the Baradian path – this means that the works are not primarily interested in the use of quantum as metaphor or analogy, as a grounding for game theory or decision theory, as an explanation of consciousness, or as a model for evolutionary biology. Rather, the ethics debate has largely accepted Barad’s terms of engagement with quantum ideas to rethink core assumptions about what the world is and how we intra-act within it (rather than analogical, model-based, or quantum-mind-driven terms of engagement). This also means that works in the quantum ethics debate are somewhat limited in terms of their technical interpretation of quantum mechanics, following Barad down a Bohr-inspired Copenhagen Interpretation instead of any one of the many other interpretations of quantum mechanics that abound in the philosophy of physics. I note the specific genesis of these ideas because – in light of Morgenthau’s ethics objection to scientism – it is all the more significant to recognize that part of Barad’s influence on the quantum ethics debate is to connect quantum ethics in IR to the implications of a particular interpretation of quantum mechanics. This is not a case of metaphors being added and stirred after the fact, but an engagement with a philosophical project that makes radical claims about the implications of the Copenhagen interpretation of quantum mechanics. Barad’s theoretical system is inseparable from the particularities of their interpretation of quantum science.

The first major work in quantum ethics in international relations was Laura Zanotti’s *Ontological Entanglements, Agency, and Ethics in International Relations*. Zanotti drew explicitly on Barad’s approach to entanglement and intra-action to overcome Cartesian dualism and Newtonian substantialism.^65^ Indeed, there is an ontological flattening that accompanies a shift from anthropocentric worldview that begins with a human interacting with objects ‘out there’ to one that starts with a phenomenon where human and nonhuman alike are always and already entangled. Ideas, importantly, are not separate from the world they purport to describe, but are part of that entangled phenomenon. To compare Cartesian-Newtonian and quantum approaches to international ethics, Zanotti contrasts the Responsibility to Protect (R2P) doctrine with the alternative community-based approach taken by a group called Partners in Health. Whereas R2P functions by applying an abstract notion to a given situation by external actors, with ‘unintended consequences’ excused as ‘the inevitable residue of ethically valid prescriptions’, Partners in Health abides a strategy of practical solidarity that recognizes the organization’s situatedness in Haitian society and responsibilities to that context.^66^ Elsewhere, Zanotti explores how this quantum perspective on entangled ethics challenges conventional abstraction-based ethical reasoning that has long held a dominant position in the Western canon.^67^ Just as Barad began from the diffractive, entangled, and fuzzy properties of quanta to reimagine social theory from the principles of quantum mechanics, Zanotti challenges the core premise of ethical debates in international relations and international political theory that abstraction and principle-based moral reasoning are appropriate philosophical practices. The connection between the system of ‘entangled ethics’ and the sources in quantum mechanics can hardly be over-emphasized – it is precisely the quantum imaginary that informs their ethical perspective.^68^

If Zanotti offers a robust reconsideration of international ethics from a largely Baradian perspective, recent works in quantum IR from Karin Fierke and Karen O’Brien are notable for the different ways in which they chart a parallel course from quantum mechanics to an ethics of entanglement. In a philosophical tour-de-force at the intersection of quantum ethics and global international relations debates, Fierke’s *Snapshots from Home* brings quantum ideas of intra-action and entangled ethics into conversation with Buddhism, Daoism, Hinduism, and the strategic thinking of Gandhi and Sun Tzu.^69^ She argues that the radical uncertainty made visible by the COVID-19 pandemic is not an aberration but the norm, and that the process of becoming ‘at home’ in the universe means accepting our place within its grandest continua – from subatomic physics to global social dynamics. O’Brien’s call for quantum social change in *You Matter More Than You Think* similarly calls for an embrace of the uncertainty and entanglement of quantum mechanics, in this case to ground a hopeful vision of an ethically responsible way of life in the face of the climate crisis. Quantum superposition provides O’Brien’s inspiration that alternatives are worth pursuing, and entanglement provides both a framework for conceptualizing our responsibility to the system as well as theorizing a mechanism for social change.^70^ These three book-length interventions reject the separation of scientific and ethical ideas and instead develop their novel theoretical models in a unified world of what Barad calls the naturalcultural. While their rejection of the science/ethics separation is grounded in a radically different logic than Morgenthau’s argument, it is remarkable how complementary the conclusions are.

The final data cluster for the ethics debate in quantum international relations consists of a recent special section of *Global Studies Quarterly* edited by Nadine Voelkner and Laura Zanotti. This section called for a ‘pause on the quest for foundations’ to instead explore the ways in which quantum approaches to international relations ‘offer interesting avenues for rethinking ethics (including scholarly research ethics) and political agency’.^71^ The editors argue that the shift from Newton to quantum – that is, a shift from assumptions of separability and causal linearity/locality to notions of entanglement, uncertainty, and complexity – render conventional ethical frameworks unfeasible. In their place, ‘ontological imaginaries associated with quantum physics open alternative ethical, political, and epistemological spaces’.^72^ While all interventions in the special section share this central premise – that a shift in ontological imaginary *demands* a new ethics, particularly because of the displacement of separability with connectedness – the forum is also notable for the variety of empirical and theoretical concerns addressed. Nadine Voelkner’s^73^ analysis of the COVID-19 virus and political responses argues that Newtonian conceptualizations of isolated viral threats (inviting warlike responses) must be replaced with a more holistic quantum ethics that foregrounds the entanglements between animals, viruses, humans, and the structural inequalities in society. Karin Fierke and Nicola Mackay^74^ take seriously the complementarity of ‘those who left’ and ‘those left behind’ as uncertain and uncomfortable opposites in refugee politics, ultimately arguing that compassionate refugee politics are only possible once we move beyond the ‘either/or potentials of life and death’. Other interventions in the special issue bring together ethical considerations relating to theoretical and methodological questions in the discipline, including the duty of scholarship to attend to the political present instead of taking sides on a for/against policy binary, the ethical status of the researcher in the context of ethnography, and an exploration of how quantum approaches to structural violence reshapes its political and ethical stakes.^75^ Although covering a range of specific topics, the special issue offers a unified contribution by centering the ethical stakes of a shift to a quantum imaginary.

The ethics debate in quantum international relations continues the provocative theoretical challenge begun with the call to develop quantum approaches. A shift from a separable world to an entangled one entails a revolutionary ethics with radical responsibility for agents in academia and the world who contribute to the continued constitution of what we understand as reality. To this end, our imaginary does not only inform the epistemological assumptions we make about knowledge production, the ontological status of what matters, and the methodological tools we use to understand the world around us. Our imaginary sets the stage for *what matters* in terms of our ethical and political systems. This connection between a specific interpretation of quantum mechanics and the quantum ethics debate in IR is also important to note because of what other paths can possibly be taken. A reader who finds that the claims made by participants in the present debate are plausible but not quite right might explore how ethical reimagining can be approached from any one of the non-Copenhagen interpretations of quantum mechanics (e.g. Many Worlds, QBism). Each of these alternative interpretations brings with it many opportunities for further engagement with rich literatures in the philosophy of physics.

## Quantum IR and Morgenthau’s ‘ethics objection’

The ethics debate is an important episode in the development of quantum international relations because it can help us to make sense of the relationship between these new approaches and established theories, theorists, and debates. In the case of the present article, the quantum ethics debate is notable because it demonstrates how an approach to International Relations theory that springs from scientific ideas can avoid the ‘ethics objection’ that Morgenthau makes against scientistic epistemology. Although the implications of this theoretical peacebuilding are metatheoretically profound, the suggestion will not be completely surprising to the careful reader of Morgenthau’s work.^76^ After all, a key element of his critique of scientistic epistemologies is that true science requires a connection to unknowability and uncertainty; there is hardly a branch of scientific thought more philosophically committed to uncertainty than quantum. Furthermore, there is a suggestion in *Scientific Man Versus Power Politics* that Morgenthau may have intended to leave his door open to quantum physics, as he is careful to add a caveat about recent developments from this work^77^: ‘The very concept of physical nature as the paradigm of reason, from which the analogy between the natural and social world derives, is invalidated by modern scientific thought itself; and it is only in rationalistic philosophy and science that it still leads a ghostlike existence’.^78^ Foreshadowing Wendt’s critique of scientific IR for not keeping up with scientific developments decades later,^79^ Morgenthau indicates in this passage an openness to sciences that remain open to uncertainty. Quantum science is not, by this definition, scientistic.

The significance of a quantum element to the ethics debate, however, extends far beyond fitting into a caveat. The ethics debate demonstrates how quantum international relations overcomes the ethics objection that Morgenthau developed vis-à-vis scientistic epistemologies. The remainder of this section considers the four elements of the ethics objection, to delineate how the ethics debate in quantum international relations overcomes this critique.

### Scientific approaches separate themselves from philosophy and ethics

The first element of the ethics objection is the lowest hurdle to overcome, but the lack of ethical consideration in much of the hardline social scientific inquiry underscores the significance of the ethics debate in quantum international relations. Just as many physicists left questions of meaning to the philosophers through the 20th century,^80^ ethical questions of relationality, positionality, complexity, and so on were often hived off from scientific paradigms of international relations and left for more theoretical schools of thought. That quantum international relations – inextricably linked with scientific concepts – has spilled so much ink at an early stage on questions of ethics demonstrate the connectedness of science and ethics. Similarly, a systematic engagement with a variety of philosophical, cosmological, and theoretical traditions though monographs of quantum international relations^81^ reveals that not only ethics but a range of philosophical perspectives are welcomed as core interlocutors of quantum thinking.

### Scientific approaches lack an understanding of power and violence

The second element of Morgenthau’s ethics objection is that truncating an understanding of the world to the limitations of rationality leaves the scientistic view unable to comprehend the exercise of power and violence. Beyond the ethics debate in quantum international relations, we can find examples of quantum approaches to theorizing power and violence. Wendt’s^82^ exploration of monadology and domination in the context of a quantum vitalist sociology provides a rich philosophical framework for theorizing power, for example, and Salter’s proposal of a quantum model of sovereignty in the Arctic challenges settler claims to sovereignty and justice.^83^ It is in the context of the ethics debate, however, that we find a sustained engagement on multiple issues relating to power and violence, from the politics of refugees to the ontology of war, and the catastrophe of nuclear weapons to the societal power of structural violence.^84^ More than a diagnosis based on principles removed from their contexts, these interventions draw directly from quantum concepts to ethical cases, and provide new ways of navigating the ethical problems explored. It is the broader significance of wave dynamics that makes the problem of structural violence difficult to address, as a phase shift of oppression does not rectify the violence,^85^ just as it is the undecidability of those who left and are left behind that demands a reimagining of modes of being and belonging.^86^ The ethics debate tackles questions of power and violence head-on, and considers them in direct connection to core conceptual models of quantum thinking.

### Separation of science from ethics leads to an ignorant and underdeveloped value system

The third element of Morgenthau’s ethics objection criticizes the separation of science from ethics because this produces an undeveloped value system. Note that it is not a claim that scientism can escape ethics, but that the ignorance of ethics produces – as if by accident – unsystematic valuations. As Barad’s^87^ reflections on diffraction demonstrate in great detail, the ‘ethics’ in quantum ethics are inextricable from the ontological foundations of quantum mechanics. That is to say, there is a deep intentionality in the engagement between science and ethics in the case of quantum international relations that develops a robust (and radical) ethics of entanglement. Zanotti’s^88^ approach to ethical reasoning is notable in this regard, as she follows Barad in developing an anti-substantialist ethics directly from the principle of entanglement. While also drawing on entangled ethics, Voelkner^89^ expands not only the scope of ethical reflection but also the scope of the scientific dialog, engaging ideas of biology, virology, and beyond to challenge the moral assumptions that served as a guide for responses to the COVID-19 pandemic. Indeed, as noted above, each contribution to the special issue of *Global Studies Quarterly* approaches the ethics debate from a position of deep engagement with quantum ideas.

### Rejection of philosophy means that scientism is not true science

The fourth and final element of the ethics objection is that the failure to engage with questions of ethics and philosophy means that scientism has lost its connection to the infinite and therefore should not be considered a true science. This element of the ethics objection is significant because it reveals most clearly Morgenthau’s view that science and philosophy ought to be linked as strategies for humanity to deal with the shock of wonderment that follows from our recognition of the infinity and unknowability of the universe. When scientism severs this connection to deeper questions that are necessarily unknowable, it then fails to live up to its ultimate purpose. The centrality of uncertainty within quantum mechanics is a strong case for these approaches to overcome this element of the ethics objection – even without ethical reflections – because uncertainty in quantum mechanics goes beyond epistemological unknowability to incorporate ontological indeterminacy. But the influential literature in the ethics debate is notable for its ongoing engagements with philosophy in addition to the embrace of ontological indeterminacy. There is not only an openness but also a sustained effort to engage with various philosophical and theoretical traditions. In addition to the diverse sources of social theory and philosophy of science that cohabit the pages of *Meeting the Universe Halfway*, Barad has delved into materialist, queer, feminist, posthumanist, and poststructuralist theories in a variety of shorter interventions.^90^ Alexander Wendt’s^91^ tour-de-force *Quantum Mind and Social Science* is deeply embedded in debates around philosophy of mind, consciousness, and social theory, in addition to scientific thought across scales of abstraction (even if it does not engage directly with considerations of the spiritual and infinite^92^). More directly connected to the ethics debate, Zanotti’s critiques of Newtonian substantialism and Kantian ethics^93^ engage widely with philosophical accounts while Fierke’s^94^ engagement with Daoist, Buddhist, and other philosophies beyond the West substantially broaden the scope of these philosophical discourses. Not only do we find evidence in these examples of an acknowledgment of uncertainty as an ontological – if technical – feature of quantum mechanics, but we also encounter rigorous engagement with theoretical thinking. A wide variety of philosophical interlocutors infuses the ethics debate with not one but many dialogs with the infinite.

This re-examination of Morgenthau’s ethics objection to scientism, within and beyond international relations, provides an important mechanism to evaluate the ethics debate in quantum international relations. In short, we can see that the engagement with ethics overcomes the four major elements of Morgenthau’s objection to scientism. But the centrality of these concerns to quantum theory tell us something even more important. It is not that quantum social theory was extended or applied to ethical questions, as may happen with other bodies of thought that are developed through particular sets of questions and then transposed to the assessment of ethical problems. Rather, in each intervention in the ethics debate of quantum international relations explores how the core concepts of quantum mechanics *demand* a reimagination of ethics alongside ontology, epistemology, methodology, metaphysics, logic, causality, temporality, and so on.

Quantum international relations thus occupies an uneasy positioning vis-à-vis familiar debates in international relations theory. The appeal to science is not made in order to belittle, marginalize, deny, or silence those approaches that might have joined the traditionalist camp in the context of the so-called Second Great Debate (but known now by names such as post-positivist, non-positivist, interpretivist, critical, poststructural, postmodern, feminist, queer, and many others). Indeed, the ontological, epistemological, methodological, metaphysical, and especially the ethical questions raised by these traditions share important common ground with many approaches within quantum international relations. A quantum imaginary renders inoperative the separations between nature and culture, scientific and humanistic thought that have led to the marginalization of consciously non-scientific perspectives. But this creates an uneasy positionality for quantum international relations and leads many within those scholarly communities to approach quantum IR with an ambivalence.^95^ By way of conclusion, I reflect on what this theoretical dialog between Morgenthau and the ethics debate can tell us about the metatheoretical status of quantum international relations within the broader field.

## Conclusion

For Hans Morgenthau, politics is a social phenomenon that demands ethical consideration and where scholars must hold a humble recognition of uncertainty. To both ends, Morgenthau finds scientistic epistemologies lacking. But what a careful reading of *Scientific Man Versus Power Politics* and *Science: Servant or Master?* reveals is that this objection to the ethical failure of scientistic epistemology is also because the false certainty goes against the ultimate aim of science itself. Quantum and relativity are both offered as examples of how science can account for uncertainty, and we might also say that these provide a connection to the wonderment that permits inquiry to sustain itself. By ignoring the transcendent and cutting away ethical considerations, scientistic epistemologies lose sight of the complexity of humanity. The ‘ethics debate’ in quantum International Relations illustrates directly how Morgenthau’s critique of scientism is not a rebuke of science itself. Indeed, contributions to quantum ethics have demonstrated that approaches drawing on scientific ideas can remain connected to considerations of human agency, uncertainty, and complexity. As scholars continue to rediscover the complexity and nuance of Morgenthau’s thought, the quantum ethics debate offers a contemporary example of why this distinction matters.

But in addition to quantum International Relations serving a useful illustrative function, I believe that there is important guidance that Morgenthau’s writings offer participants in the quantum IR debates as a form of guardrails against the excesses of scientism. First, the danger set out in especially clear terms in *Scientific Man Versus Power Politics*, is that scientific framings in social inquiry have in the past led to claims of certainty that a theory cannot hope to bear out in practice. Although the quantum ethics debate has largely avoided direct policy commentary, as the broader quantum IR community bridges the gap to the world of policy it is important to avoid associations of the scientific provenance of quantum ideas with promises of higher degrees of certainty. The language may be more appropriate to certain contexts, but that does not negate the appropriateness of other frameworks to other contexts. The danger of promising certainty is especially important in quantum IR given the central role of uncertainty and complexity in quantum theory itself.

A second point of advice to draw is that to capture a broader range of human experience, science-related approaches like quantum IR must ensure that an ongoing dialog with humanistic elements is maintained. Although I have discussed above the ways in which the quantum ethics debate maintains this dialog with the infinite that Morgenthau finds lacking in scientism, it is important to recognize that this conversation has been rather limited in the scope of its engagement with history and philosophy of science, on the one hand, and international ethics, on the other. Because of the influence of Barad and Wendt on quantum IR, much of the conversation generally abides the Copenhagen interpretation of quantum mechanics; this leaves many unturned stones in the provocative other paths exploring the meaning of quantum. Furthermore, the quantum ethics debate demonstrates that the effectiveness of quantum social theory in raising ethical questions opens a number of potential conversations with existing work in international ethics. Quantum IR scholars might recognize that these opportunities exist in the many different research areas that quantum perspectives might consider in future. Further research in quantum IR should endeavor a broader dialog with philosophy of physics to incorporate different interpretations of quantum mechanics, and with existing International Relations scholarship. In the specific case of ethics, concepts of contradiction and complementarity would be especially powerful in undoing simple right/wrong binaries, but also raise important questions about what ethical action looks like when acts are both right and wrong; many worlds models or information-centric interpretations like QBism would provide further fodder for the consideration of what it means to act ethically.

A first step toward heeding the advice of Morgenthau is found in this article itself. By engaging with Morgenthau’s own writings on science, we uncovered the relationship between ethics and epistemology that is broken within a scientistic epistemology. In the case of the ethics debate in quantum IR, we found a clear example of how scientific ideas can nevertheless directly inform ethical discussions. Significantly, we noted how this epistemology/ethics relationship can be rediscovered by a scientific perspective that recognizes its own limits and opens new conceptual space for further debate. In addition to illustrating Morgenthau’s distinction between true science and scientism, the article also offers a model of how quantum International Relations can engage with existing debates on the metatheory of International Relations.

## Notes

1. Hans J. Morgenthau, *Scientific Man Versus Power Politics* (Chicago, IL: University of Chicago Press, 1965), p.132. Henceforth *SMVPP*.

2. For more on the connection of Morgenthau and contemporaries to quantum theory, see Jayson C. Waters, ‘Mind, Matter, and Motion: A Genealogy of Quantum Entanglement and Estrangement’, in James Der Derian and Alexander Wendt (eds), *Quantum International Relations: A Human Science for World Politics* (New York, NY: Oxford University Press, 2022), pp. 49–69; Jayson C. Waters, ‘Estranged/Entangled: The History, Theory, and Technology of Quantum Mechanics in International Relations’ (PhD Thesis, University of Sydney, Australia, 2022).

3. James Der Derian and Alexander Wendt, ‘‘Quantizing International Relations’: The Case for Quantum Approaches to International Theory and Security Practice’, *Security Dialogue*, 51(5), 2020, p. 409.

4. Alexander Wendt, *Quantum Mind and Social Science: Unifying Physical and Social Ontology* (New York, NY: Cambridge University Press, 2015); James Der Derian, ‘Quantum Diplomacy, German–US Relations and the Psychogeography of Berlin’, *The Hague Journal of Diplomacy*, 6(3–4), 2011, pp. 373–92; James Der Derian, ‘From War 2.0 to Quantum War: The Superpositionality of Global Violence’, *Australian Journal of International Affairs* 67(5), 2013 pp. 570–85.

5. Laura Zanotti, *Ontological Entanglements, Agency and Ethics in International Relations: Exploring the Crossroads* (London: Routledge, 2018); Karen O’Brien, *You Matter More Than You Think: Quantum Social Change for a Thriving World* (Oslo: cChange Press, 2021).

6. Alexander Wendt, ‘Why IR Scholars Should Care About Quantum Theory, Part II: Critics in the PITs’, *International Theory*, 14(1), 2022, pp. 194–209.

7. Italo Brandimarte, ‘Subjects of Quantum Measurement: Surveillance and Affect in the War on Terror’, *International Political Sociology*, 16(3), 2023, pp. 1–27; Karin M. Fierke and Francisco Antonio-Alfonso, ‘Language, Entanglement and the New Silk Roads’, *Asian Journal of Comparative Politics*, 3(3), 2018, pp. 194–206.

8. Patrick Thaddeus Jackson, ‘Fundamental Grounding’, *Perspectives on Politics*, 14(4), 2016, pp. 1153–7; Bentley B. Allan, ‘Social Action in Quantum Socail Science’, *Millennium*, 47(1), 2018, pp. 87–98; Laura Sjoberg, ‘Quantum Ambivalence’, *Millennium*, 49(1), 2020, pp. 126–39.

9. This was already recognized by authors of the time, e.g., Vital 1967, 554.

10. Robert A. Dahl, ‘The Behavioral Approach in Political Science: Epitaph for a Monument to a Successful Protest’, *American Political Science Review*, 55(4), 1961, pp. 763–72.

11. Arend Lijphart, ‘The Structure of the Theoretical Revolution in International Relations’, *International Studies Quarterly*, 18(1), 1974, 68*ff*.

12. J. David Singer, ‘The Behavioral Science Approach to International Relations: Payoff and Prospect’, *SAIS Review*, 10(4), 1996, p. 18.

13. Hedley Bull, ‘International Theory: The Case for a Classical Approach’, *World Politics*, 18(3), 1966, p. 372.

14. Bull, ‘International Theory’.

15. Hans J. Morgenthau, ‘Common Sense and Theories of International Relations’, *Journal of International Affairs*, 21(2), 1967, p. 210.

16. While Bull pulls no punches in criticizing the density of Kaplan’s prose, Kaplan is ruthless in his response to traditionalist critiques: “The traditionalists are often quite intelligent and witty people. Why then do they make such gross mistakes? Surely there must be some- thing seriously wrong with an approach that devotes so much effort to such ill-informed criticism. One suspects that this sorry product is the consequence of the traditionalist view of philosophy as elegant but undisciplined speculation-speculation devoid of serious substantive or methodological concerns. Thus traditionalists repeat the same refrain like a gramophone endlessly playing a single record; that refrain is beautifully orchestrated, wittily produced, and sensitive only to the wear of the needle in the groove.” See Morton Kaplan, ‘The New Great Debate: Traditionalism vs. Science in International Relations’, *World Politics*, 19(1), 1966, p. 20.

17. Stanley Hoffmann, ‘An American Social Science: International Relations’, *Deadalus*, 106(3), 1977, pp. 41–60.

18. Kalevi J. Holsti, *The Dividing Discipline: Hegemony and Diversity in International Theory* (London: Unwin Hyman, 1985), p. 39.

19. Martin Hollis and Steve Smith, *Explaining and Understanding in International Relations* (New York, NY: Oxford University Press, 1990); Robert O. Keohane, ‘International Institutions: Two Approaches’, *International Studies Quarterly*, 32(4), 1988, pp. 376–96; Yosef Lapid, ‘The Third Great Debate: On the Prospects of International Theory in a Post-Positivist Era’, *International Studies Quarterly*, 33(3), 1989, pp. 235–54.

20. Morgenthau, *SMVPP*.

21. The origin of the work is also unique, beginning as a forgotten manuscript drafted in the 1930s, developed through invited lectures in the early 1960s, and published only in 1972. Hans J. Morgenthau, *Science: Servant or Master?* (New York, NY: New American Library, 1972), p. xxi. Henceforth *SSOM*.

22. Michael C. Williams, ‘Why Ideas Matter in International Relations: Hans Morgenthau, Classical Realism, and the Moral Construction of Power Politics’, *International Organization*, 58(4), 2004, p. 652n73.

23. Wiliam Scheuerman, *Morgenthau* (Malden: Polity, 2007), p. 6; Michael C. Williams, *Realism Reconsidered: The Legacy of Hans Morgenthau in International Relations* (New York, NY: Oxford University Press, 2007).

24. Sean Molloy, *The Hidden History of Realism: A Genealogy of Power Politics* (London: Palgrave Macmillan, 2006), p. 79.

25. Felix Rösch, ‘The Human Condition of Politics: Considering the Legacy of Hans J. Morgenthau for International Relations’, *Journal of International Political Theory*, 9(1), 2013, pp. 1–21. See also Felix Rösch, *Power, Knowledge, and Dissent in Morgenthau’s Worldview* (London: Palgrave Macmillan, 2015), esp. chs. 3, 5.

26. I note Sean Molloy as a reader of Morgenthau who has previously drawn important attention to *SSOM*. For example, Sean Molloy, ‘Truth, Power, Theory: Hans Morgenthau’s Formulation of Realism’, *Diplomacy and Statecraft*, 15, 2004, pp. 1–34.

27. See Michael C. Williams, *The Realist Tradition and the Limits of International Relations* (New York, NY: Cambridge University Press, 2005), p, 94. However, this comes as no surprise given that the work began as a lecture-length critique of liberalism, which Morgenthau himself notes (Morgenthau, *SMVPP*, p. v).

28. Edward Hallett Carr, *The Twenty Years’ Crisis, 1919-1939: An Introduction to the Study of International Relations* (New York, NY: Perennial, 1965), ch. 4; Morgenthau, *SMVPP*, p. 75 and passim.

29. Morgenthau, *SMVPP*, p. 5.

30. Morgenthau, *SMVPP*, p. 18.

31. Morgenthau, *SMVPP*, pp. 16–17

32. Morgenthau, *SMVPP*, pp. 27–28, 95.

33. “The age is forever searching for the philosopher’s stone, the magic formula, which, mechanically applied, will produce the desired result and thus substitute for the uncertainties and risks of political action the certitude of rational calculation. Since, however, what the seekers after the magic formula want is simple, rational, mechanical, and what they have to deal with is complicated, irrational, incalculable, they are compelled, in order to present at least the semblance of scientific solutions, to simplify the reality of international politics and to develop what one might call the ‘method of the single cause.’” Morgenthau, *SMVPP*, p. 95.

34. Morgenthau, *SMVPP*, p. 168.

35. Morgenthau, ‘Common Sense’, pp. 170, 185.

36. Morgenthau, ‘Common Sense’, p. 201.

37. Morgenthau, *SMVPP*, p. 101.

38. Molloy, ‘Truth, Power, Theory’, p. 2.

39. Morgenthau, *SSOM*, p. 1.

40. Morgenthau, *SSOM*, p. 44.

41. Morgenthau, *SSOM*, p. 20.

42. Morgenthau, *SSOM*, p.44.

43. Morgenthau, *SSOM*, p. 46.

44. Morgenthau, *SSOM*, p. 13. For more on immanence and transcendence in Morgenthau’s view of science, see Felix Rösch, ‘Crisis, Values, and the Purpose of Science: Hans Morgenthau in Europe’, *Ethics & International Affairs*, 30(1), 2016, pp. 23–31.

45. Morgenthau, *SSOM*, pp. 6–7.

46. Morgenthau, *SSOM*, p. 13.

47. Morgenthau, *SSOM*, pp. 16–7.

48. Morgenthau, *SSOM*, p. 13.

49. This recalls the argument of Arnold Wolfers that moral philosophy is inescapable. See Arnold Wolfers, *Discord and Cooperation: Essays on International Politics* (Baltimore, MD: Johns Hopkins University Press, 1962), pp. xiv, 237.

50. Morgenthau, *SSOM*, pp. 60, 66.

51. Morgenthau, *SSOM*, p. 61.

52. See the introduction and Morgenthau, *SMVPP*, pp. 131–9.

53. Morgenthau, *SSOM*, p. 10.

54. Morgenthau, *SSOM*, p. 62; see also Molloy on the possibility of a “true” science, Molloy, *The Hidden History*, pp. 79–80.

55. Morgenthau, *SSOM*, p. 5.

56. Zanotti, *Ontological Entanglements*.

57. Nadine Voelkner and Laura Zanotti, ‘Ethics in a Quantum World’, *Global Studies Quarterly*, 2(3), 2022, pp. 1–5.

58. Karen Barad, *Meeting the Universe Halfway: The Englement of Matter and Meaning* (Durham, NC: Duke University Press, 2007), pp. 7, 25.

59. Barad, *Meeting the Universe Halfway*, p. 119.

60. Barad, *Meeting the Universe Halfway*, pp. 136–7, 197.

61. Barad, *Meeting the Universe Halfway*, p. 382.

62. Barad, *Meeting the Universe Halfway*, p. 353.

63. More recently, Barad (2017; 2019) has applied this thought to questions of nuclear politics, uncovering the connection between space, time, and how politics and physics come to matter. See Karen Barad, ‘Troubling Time/s and Ecologies of Nothingness: Re-turning, Re-membering and Facing the Incalculable’, *New Formations*, 92, 2017, pp. 56–82; Karen Barad, ‘After the End of the World: Entangled Nuclear Colonialisms, Matters of Force, and the Material Force of Justice’, *Theory & Event*, 22(3), 2019, pp. 524–50.

64. Notably, their influence can be identified beyond quantum international relations debates through literatures on feminist science and technology studies and critical security studies. See Claudia Aradau, ‘Security that Matters: Critical Infrastructure and Objects of Protection’, *Security Dialogue*, 41(5), 2010, pp. 491–514; Vicky Squire, ‘Reshaping Critical Geopolitics? The Materialist Challenge’, *Review of International Studies*, 41(1), 2015, pp. 139–59; Delf Rothe, ‘Jellyfish Encounters: Science, Technology, and Security in the Anthropocene Ocean’, *Critical Studies on Security*, 8(2), 2020, pp. 145–59; Lucy Suchman, ‘Algorithmic Warfare and the Reinvention of Accuracy’, *Critical Studies on Security*, 8(2), 2020, pp. 175–87.

65. Zanotti suggests that it is this anti-Cartesian ethos present in Barad that differentiates the project from Wendt’s approach to quantum consciousness. See Zanotti, *Ontological Entanglements*, chs 3–4. On the Wendt/Barad disagreement, see also Kimberly Hutchings, ‘Empire and Insurgency: The Politics of Truth in Alexander Wendt’s *Quantum Mind and Social Science: Unifying Physical and Social Ontology*’, *International Theory*, 14(1), 2022, pp. 183–92.

66. Zanotti, *Ontological Entanglements*, chs. 8–9.

67. Laura Zanotti, ‘Decolonizing the Political Ontology of Kantian Ethics: A Quantum Perspective’, *Journal of International Political Theory*, 17(3), 2021, pp. 448–67.

68. Critical accounts of quantum IR have raised the point that it is sometimes possible to combine different concepts into a mixture that reaches a quantum conclusion without proceeding from quantum concepts. While these ad hoc combinations may be possible in some cases, the existence of “back roads” does not disprove the highway. We might, in line with Wendt (2015), call this ability to address a large volume of conceptual problems in one fell swoop the “elegance” of quantum theory. Further, the possibility of assembling non-quantum paths does not challenge that the quantum path emerged from quantum social theory.

69. Karin M. Fierke, *Snapshots from Home: Mind, Action, and Strategy in an Uncertain World* (Bristol: Bristol University Press, 2022).

70. O’Brien, *You Matter More Than You Think*.

71. Voelkner and Zanotti, ‘Ethics in a Quantum World’, p. 2.

72. Voelkner and Zanotti, ‘Ethics in a Quantum World’, p. 2.

73. Nadine Voelkner, ‘Viral Becomings: From Mechanical Viruses to Viral (Dis)Entanglements in Preventing Global Disease’, *Global Studies Quarterly*, 2(3), 2022, pp. 1–12.

74. Karin M. Fierke and Nicola Mackay, ‘Those Who Left/Are Left Behind: Schrodinger’s Refugee and the Ethics of Complementarity’, *Global Studies Quarterly*, 2(3), 2022, pp. 1–12.

75. Chris McIntosh, ‘From Policy Relevance to Present Relevance: Entanglement, Scholarly Responsibility, and the Ethics of Quantum Social Theory’, *Global Studies Quarterly*, 2(3), 2022, pp. 1–12; Liberty Chee, ‘Being of Use: Diffraction and an Ethics of Truth-Telling in Post-Cartesian IR’, *Global Studies Quarterly*, 2(3), 2022, pp. 1–10; Michael P. A. Murphy, ‘Violent Interference: Structural Violence, Quantum International Relations, and the Ethics of Entanglement’, *Global Studies Quarterly*, 2(3), 2022, pp. 1–12.

76. E.g., Molloy, ‘Truth, Power, Theory’; Molloy, *The Hidden History*; Rösch, ‘Crisis, Values, and the Purpose of Science’; Rösch, *Power, Knowledge, and Dissent*.

77. *SMVPP* appeared on the heels of a trio of quantum-related Nobel Prizes, including Wolfgang Pauli in 1945, Isidor Isaac Rabi in 1944, and Otto Stern in 1943. Stern’s Nobel Prize-winning experiment with Walther Gerlach has been the subject of theoretical debate in quantum social theory. See Barad, *Meeting the Universe Halfway*; Michael P. A. Murphy, ‘Entangled Observers? A Quantum Perspective on Authority in Critical Security Studies’, *Critical Studies on Security*, 10(1), 2022, pp. 119–31.

78. Morgenthau, *SMVPP*, p. 126.

79. Wendt, *Quantum Mind and Social Science*.

80. David Mermin disapprovingly summarized this attitude as “shut up and calculate: ‘What’s Wrong with This Pillow?’, *Physics Today*, 42(4), 1989, p. 9.

81. Wendt, *Quantum Mind and Social Science*; Zanotti, *Ontological Entanglements;* Michael P. A. Murphy, *Quantum Social Theory for Critical International Relations Theorists: Quantizing Critique* (London: Palgrave Macmillan, 2021); Fierke, *Snapshots from Home.*

82. Wendt, *Quantum Mind and Social Science*, ch. 14.

83. Mark B. Salter, ‘Quantum Sovereignty + Entanglement’, in James Der Derian and Alexander Wendt (eds), *Quantum International Relations: A Human Science for World Politics* (New York, NY: Oxford University Press, 2023), pp. 262–80.

84. Fierke and Mackay, ‘Those Who Left’; Chris McIntosh, ‘Writing Quantum Entanglement into International Relations: Temporality, Positionality, and the Ontology of War’, *Millennium*, 49(1), 2021, pp. 162–74; Barad, ‘Troubling Time/s’; Barad, ‘After the End of the World’; Murphy, ‘Violent Interference’.

85. Murphy, ‘Violent Interference’.

86. Fierke and Mackay, ‘Those Who Left’.

87. Barad, *Meeting the Universe Halfway*; Karen Barad, ‘Diffracting Diffraction: Cutting Together-Apart’, *Parallax*, 20(3), 2014, pp. 168–87.

88. Zanotti, *Ontological Entanglements*; Zanotti, ‘Decolonizing the Political Ontology’.

89. Voelkner, ‘Viral Becomings’.

90. E.g., Barad, *Meeting the Universe Halfway*; Barad, ‘Diffracting Diffraction’; Karen Barad, ‘On Touching—The Inhuman That Therefore I Am’, *Differences*, 23(3), 2010, pp. 206–33.

91. Wendt, *Quantum Mind and Social Science*.

92. J. Peter Burgess, ‘Science Blurring its Edges into Spirit: The Quantum Path to Ātma’, *Millennium*, 47(1), 2018, pp. 128–41.

93. Zanotti, *Ontological Entanglements*; Zanotti, ‘Decolonizing the Political Ontology’.

94. Fierke, *Snapshots from Home*; Karin M. Fierke, ‘Consciousness at the Interface: Wendt, Eastern Wisdom, and the Ethics of Intra-Action’, *Critical Review*, 29(2), 2017, pp. 141–69.

95. Sjoberg, ‘Quantum Ambivalence’.

